# Sex-related variation in compact bone microstructure of the femoral diaphysis in juvenile rabbits

**DOI:** 10.1186/1751-0147-50-15

**Published:** 2008-06-03

**Authors:** Monika Martiniaková, Radoslav Omelka, Birgit Grosskopf, Alexander V Sirotkin, Peter Chrenek

**Affiliations:** 1 Department of Zoology and Anthropology, Constantine the Philosopher University, Nábrežie mládeže 91, 949 74 Nitra, Slovak Republic; 2 Department of Botany and Genetics, Constantine the Philosopher University, Nábrežie mládeže 91, 949 74 Nitra, Slovak Republic; 3 Johann Friedrich Blumenbach Institute of Zoology and Anthropology, Georg-August University, Bürgerstrasse 50, 37 073 Göttingen, Germany; 4 Slovak Agricultural Research Centre, Hlohovská 2, 949 92 Nitra, Slovak Republic

## Abstract

**Background:**

While gross morphological changes in the skeleton between males and females are well know, differences between sexes in the histomorphology are less known. It is important to have knowledge on the bone structure of rabbits, as this is a widely used species in biomedical research. A study was performed to evaluate the association between sex and the compact bone morphology of the femoral diaphysis in juvenile rabbits.

**Methods:**

Seventeen clinically healthy 2–3 month-old rabbits (9 females, 8 males) were included in the study. The rabbits were euthanized and the right femur was sampled for analysis. 70–80 microns thick bone sections of the femoral diaphysis were prepared using standard histological equipment. The qualitative histological characteristics were determined according to internationally accepted classification systems while the quantitative parameters were assessed using the software Scion Image. Areas, perimeters, minimum and maximum diameters of primary osteons' vascular canals, Haversian canals and secondary osteons were measured. Additionally, blood plasma concentrations of progesterone, corticosterone, IGF-I, testosterone and estradiol were analyzed.

**Results:**

Qualitative histological characteristics were similar for both sexes. However, variations of certain quantitative histological characteristics were identified. Measured parameters of the primary osteons' vascular canals were higher in males than for females. On the other hand, females had significant higher values of secondary osteons parameters. Differences in Haversian canals parameters were only significant for minimum diameter.

**Conclusion:**

The study demonstrated that quantitative histological characteristics of compact bone tissue of the femoral diaphysis in juvenile rabbits were sex dependent. The variations may be associated with different growth and modeling of the femur through influence by sex-specific steroids, mechanical loads, genetic factors and a multitude of other sources. The results can be applied in experimental studies focusing on comparison of the skeletal biology of the sexes.

## Background

Animal models are commonly used in the study of skeletal biology. The rabbit is one of the most commonly used animals for biomedical research, being used in approximately 35% of musculoskeletal research studies [1]. This is in part due to ease of handling and size, but the rabbit is also convenient as it reaches skeletal maturity shortly after sexual maturity at around 6 months of age [2,3].

In general, compact bone tissue forms the shafts of long bones, the surfaces of their extremities, short bones, and the outer and inner layer of the cranial vault in rabbits. Basic constituents of its structural organization are primary and secondary osteons (Haversian systems). Primary osteons are not surrounded by a reversal (cement) line and their lamella merge smoothly with the surrounding bone, while secondary osteons consist of a central (Haversian) canal surrounded by concentric rings (lamellae) of matrix [4,5].

Histological research of compact bone tissue can be carried out qualitatively and quantitatively. With the qualitative approach, the structural pattern of the bone tissue is identified, while osteons and/or Haversian canals are counted and measured by the quantitative approach.

On the macroscopic level, sexual dimorphisms are clearly manifested in the skeleton. Bones of males are generally bigger and more robust than those of females. Other more specific differences are a consequence of different adaptation. For example, the male pelvis is primarily adapted to striding whereas the female pelvis displays size and shape differences connected to giving birth [6]. Unlike macroscopic differences between the sexes, the microscopic ones are not unambiguously characterized. Moreover, most studies describing sex-related variations in compact bone have focused on humans, while only a few studies have been carried out in animals.

Histological studies focused on adult humans revealed no significant differences in osteon and Haversian canal area between the sexes [7]. On the other hand, Haversian canal size [8] and osteon size [9] were greater in females (*P *< 0.05). It was found while osteon size increases with age among females, it decreases in males. According to Mulhern and Van Gerven [10], males have significantly more intact osteons than females, whereas females dispose significantly larger osteons than males. On the other hand, significant differences in the secondary osteon area for adult male and female *rhesus macaques *were observed [11](*P *< 0.05). Male macaques had larger Haversian systems than their female counterparts, and this difference has been attributed to the overall larger body size of the male macaques. In a study by Urbanová and Novotný [12], no significant differences between the sexes were detected in the Haversian canals and secondary osteons parameters from various bones of adult cattle (*Bos taurus*), pigs (*Sus scrofa domestica*), sheep (*Ovis aries*) and horses (*Equus caballus*). However, only two individuals from each species were analysed. In some research cited above, statistically significant differences in the microstructure of compact bone tissue were found between the sexes for adult humans and/or animals. It is not clear, however, whether the differences are present in juvenile individuals as well, nor how these differences change during an individual's ontogenesis including puberty.

In our study we used rabbits as an animal model to investigate in detail the microscopic structure of compact bone between juvenile females and males. The microstructure of the femoral diaphysis was evaluated based on qualitative and quantitative histological characteristics. The concentrations of selected hormones related to growth and development in blood plasma were also assessed.

## Methods

### Animals

Seventeen clinically healthy 2–3 month-old rabbits (*Oryctolagus cuniculus*, New Zealand White breed) (mean age 77 day) were used. The group consisted of 9 females and 8 males with mean bodyweights of 2.482 kg and 2.623 kg, respectively. The animals were obtained from an experimental farm of the Slovak Agricultural Research Centre (SARC), Nitra, Slovakia. They were housed in individual flat-deck wire cages (area 0.34 m^2^), under a constant photoperiod of 14 h of day-light. Daily physical activity was restricted by the cage size. The temperature (18–20°C) and humidity (65%) of the building were recorded continually by means of a thermograph positioned at the same level as the cages. The rabbits were fed *ad libitum *with a commercial diet (KKV, Refka, s. r. o., Rastislavice, Slovakia), and water was provided *ad libitum *with nipple drinkers. The concentrations of selected elements in the diet were: Ca 8.18 g.kg^-1^, P 5.45 g.kg^-1^, Mg 0.88 g.kg^-1^, Na 1.44 g.kg^-1^, K 9.57 g.kg^-1^, Fe 289.55 g.kg^-1^, Zn 0.63 g.kg^-1^. The rabbits were kept and euthanized especially for other investigations (e.g., histological analyses of internal organs, cytogenetic analyses of blood cells) at SARC. These experiments, however, did not influence bone metabolism of the animals. All procedures were approved by the Animal Experimental Committee of the Slovak Republic.

### Procedures

The animals were euthanized by electrocution and bone specimens were obtained during necropsy. Only the right femur of the 17 rabbits was analyzed. Each of the bones was sectioned at the midshaft of its diaphysis, where the compact bone is thick and provides a large area for study of the bone microstructure. One transversal section was taken of each femur. The isolated segments were macerated and degreased [13] followed by embedding in epoxy resin (Biodur, Günter von Hagens, Heidelberg, Germany). Transverse sections (70–80 μm) were prepared with a sawing microtome Leitz 1600 (Leica, Wetzlar, Germany) and affixed to glass slides by Eukitt (Merck, Darmstadt, Germany). The qualitative histological characteristics were determined according to the classification systems by Enlow and Brown [14] and Ricqlès *et al. *[15]. The quantitative parameters were assessed using the software Scion Image (Scion Corporation, Maryland, USA) in anterior, posterior, medial and lateral views of thin section.

Since osteon size may vary in the different views [16], we measured area, perimeter, and the minimum and maximum diameter of primary osteons' vascular canals, Haversian canals and secondary osteons in all views of thin section in order to minimize the difference in the individual in the statistics. At least 20 vascular canals of primary osteons were measured in each individual (5 each from anterior, posterior, medial and lateral views). On the other hand, all secondary osteons, which were not in a resorption phase and could clearly be outlined using the software Scion Image were measured. Before measuring, the sections were carbonized [17]. The older osteons got lighter, the younger ones got darker, and the reversal lines of secondary osteons became much more visible. Secondary osteons were distinguished from primary osteons (i.e., primary vascular canals) on the basis of the well defined peripheral boundary (cement line) between the osteon and the surrounding tissue.

### Hormonal analyses

The concentration of progesterone, corticosterone, IGF-I, testosterone and estradiol was determined in 20–50 μl plasma using RIA/IRMA kits (DSL, Webster, Texas, USA) according to the manufacturer's instructions. The characteristics of the assay have been described elsewhere [18].

### Statistics

The quantitative histological characteristics were analyzed by the Student's t-test. Hormonal concentrations were analyzed by Duncan test using the computer programs SigmaStat and SigmaPlot 9.0. (Systat Software, Inc., Erkrath, Germany). *P*-values less than 0.05 were considered significant.

## Results

### Qualitative histological characteristics

**Figure 1:**
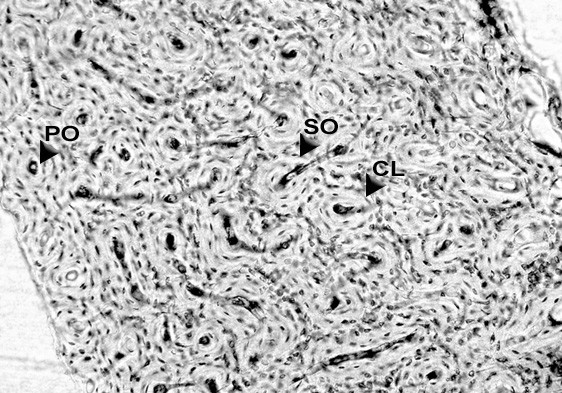
Bone tissue microstructure of a juvenile male rabbit. PO – primary osteon. SO – secondary osteon. CL – cement line. Femur, diaphysis. Magnification × 100.

The femoral diaphysis had a similar morphology despite the sex of the rabbits. A primary vascular longitudinal bone tissue formed the inner layer surrounding the medullary cavity and also the periosteal surface of the bone. This type of bone was created by vascular canals, which ran in a direction essentially parallel to the long axis of the bone (Fig. 1). In the middle parts of the compacta, a layer of dense Haversian bone tissue followed. This layer consisted of many secondary osteons. Further, toward the periosteal surface the Haversian bone tissue was gradually replaced by primary vascular radial, but predominantly longitudinal bone tissue.

### Quantitative histological characteristics

Four hundred and three vascular canals of primary osteons, 145 Haversian canals and 145 secondary osteons were measured (Table 1). For the primary osteons' vascular canals, area (*P *< 0.05), perimeter (*P *< 0.01) and maximum diameter (*P *< 0.01) had significantly higher values in males than in females. On the other hand, females had higher values for the secondary osteons parameters and Haversian canals. The differences for all parameters of the secondary osteons were statistically significant (*P *< 0.01; *P *< 0.001), but for Haversian canal parameters only the minimum diameter was different (*P *< 0.05).

**Table 1:** Measured parameters of vascular canals of primary osteons, Haversian canals and secondary osteons in the femoral diaphysis of 9 female and 8 male juvenile rabbits.

**Structure**	**Parameters**	**Sex**	**Mean**	**SD**	***P*-value**
Vascular canals of primary osteons	area (μm^2^)	females	173.16	64.63	0.016*
		males	194.81	54.44	
	perimeter (μm)	females	41.27	7.87	0.004*
		males	44.47	7.11	
	max. diameter (μm)	females	20.10	4.64	0.005*
		males	21.98	4.54	
	min. diameter (μm)	females	6.74	1.44	0.132
		males	7.05	1.37	
Haversian canals	area (μm^2^)	females	282.93	86.83	0.063
		males	243.09	66.27	
	perimeter (μm)	females	54.51	9.83	0.256
		males	51.66	9.20	
	max. diameter (μm)	females	27.00	5.94	0.612
		males	26.22	5.73	
	min. diameter (μm)	females	8.25	1.63	0.016*
		males	7.31	0.89	
Secondary osteons	area (μm^2^)	females	9470.55	2173.71	0.0002*
		males	7224.77	2472.21	
	perimeter (μm)	females	481.15	65.51	0.0001*
		males	411.13	72.25	
	max. diameter (μm)	females	231.38	44.82	0.0091*
		males	199.83	49.64	
	min. diameter (μm)	females	52.69	9.79	0.0011*
		males	44.76	6.60	

SD: standard deviation, * : *P *< 0.05

### Concentrations of selected hormones

RIA revealed substantial levels of progesterone, corticosterone, IGF-I, testosterone and estradiol in the blood plasma of all animals (Table 2). Significant differences between the sexes were observed only for testosterone and estradiol (*P *< 0.05).

**Table 2:** Mean concentration of hormones in the blood plasma of 9 female and 8 male juvenile rabbits.

**Sex**	**Progesterone (ng/ml)**	**Corticosterone (ng/ml)**	**IGF-I (ng/ml)**	**Testosterone (ng/ml)**	**Estradiol (ng/ml)**
Female	2.74 ± 0.44	67.87 ± 6.26	43.33 ± 5.66	1.98 ± 0.21	6.43 ± 0.31*
Male	2.24 ± 1.61	55.95 ± 13.99	47.92 ± 12.76	2.93 ± 0.32*	5.21 ± 0.26

* : *P *< 0.05

## Discussion

In general, our qualitative histological results correspond with those previously reported [19,20]. The basic structural pattern of compact bone tissue was primary vascular longitudinal in both sexes of juvenile rabbits. In addition, dense Haversian bone tissue and/or primary vascular radial bone tissue were found in the middle part of the compacta. In humans, qualitative characteristics of compact bone tissue are independent of sex [7-9]. This demonstrates that qualitative bone morphology expresses a low sex-related variation. Together with a strong genetic component of the qualitative traits, it allows them to be used for taxonomic identification of various species [5,19].

The quantitative histological characteristics were comparable with those previously observed in rabbits [20] and the values for the mean diameter of secondary osteons were similar to those of hare [17]. On the other hand, mean diameters of Haversian canals were higher than found in rabbits [21]. However, these authors did not mention which bone they examined.

The present study demonstrated significant difference in the quantitative histological characteristics between the sexes as males had larger primary osteons' vascular canals than females. This observation may be explained by the different length of the femur in males vs. females (males: 9.5 cm, females: 9.1 cm). Martiniaková *et al. *[5] found that values of the primary osteons' vascular canals were higher in individuals with longer bones than in those having shorter bones. In females, significant higher values of the Haversian canals and secondary osteons parameters were observed. It indicates that males can accommodate fewer large secondary osteons than small osteons per square millimeter of compact bone tissue compared to females. That females have higher values for the secondary osteons has also been described in humans [9,10]. In general, remodeling of bones seems to be lower in females, since estrogens slow down bone remodeling and protect against bone loss [22].

The processes of bone growth and modeling are typical for all bones of juvenile individuals. During growth, bone formation necessarily precedes bone resorption [23]. In the processes of bone growth and modeling, the sex-specific steroids (estrogens in females and androgens in males) play an important role. The bone-sparing effect of estrogen is mainly related to its ability to block bone resorption, although stimulation of bone formation is likely to play a contributory role [24]. Also, androgens have region-specific actions on bone growth and remodeling leading to larger bones in males [25,26].

The hormone analyses in the present study confirm previous reports of the presence of corticosterone [27] and gonadal steroids [28,29] in the plasma of adult rabbits. Significant differences between the sexes were observed only for testosterone and estradiol. It is known that hormones do not act on bone tissue in isolation [30]. Bone tissue must integrate a variety of signals that result from mechanical loads, hormones, and a multitude of other sources. The effects of different mechanical loads as well as other sources of environmental factors were limited in our study by standardization of keeping (housing) conditions of the animals. Finally, bone microstructural morphology is to some degree genetically mediated, and provides a direction for further exploration into this phenomenon [11]. Beamer *et al. *[31] mention that environmental factors are important contributors to variability in bone microstructure also between the sexes, but genetics play a substantial role, with estimates of heritability ranging from 40–93%. We believe that the described variations in quantitative histological characteristics of compact bone tissue could be caused by different growth and modeling of the femora through sex-specific steroids, mechanical loads, genetic factors and a multitude of other sources.

Our results were based on examination of thin sections taken from a limited area of the femoral diaphysis. Therefore, there are limitations of extrapolating the findings to the entire femur or femoral diaphysis. Bone remodeling is namely differently distributed along the diaphysis. It is known that the remodeling activity is generally greater in endosteal regions than in periosteal regions. For this aim, increased prevalence of osteonal variants is found near the medullary canal (i.e., endosteal regions) than near the periosteum. In addition, the angle of section influences the morphometric data. The angle of section was not recorded in our study. On the other hand, the influence of the angle of section is reduced by the number of osteons examined. In our study, at least 20 vascular canals of primary osteons and all secondary osteons were measured in each individual. Despite a number of limitations arising from this study, the results can be applied in experimental studies focusing on comparison of the midshaft of femoral diaphysis between the sexes.

## Conclusion

The study demonstrated that quantitative histological characteristics of compact bone tissue of the femoral diaphysis in juvenile rabbits were sex dependent. Specifically, area, perimeter and maximum diameter of the vascular canals of primary osteons were higher in males. Females had significant higher values of the secondary osteons parameters. Differences in Haversian canals parameters were only significant for minimum diameter. We believe that the variations may be associated with different growth and modeling of the femur through influence by sex-specific steroids, mechanical loads, genetic factors and a multitude of other sources. The effect of sex-specific hormones on sex-related variation in compact bone tissue seems to be important also in juvenile rabbits. Our study seems to be the first reporting differences in compact bone tissue between juvenile females and males rabbits. The results can be applied in experimental studies focusing on comparison of the skeletal biology of the sexes.

## Competing interests

The authors declare that they have no competing interests.

## Authors' contributions

MM carried out the histological analysis of examined bones, RO performed the statistical analysis of presented data, BG prepared thin sections for histological analysis, AS carried out the hormonal analysis, PCh supported animal care and femora taking. All authors read and approved the final manuscript.
